# Genome-Wide Identification and Comparative Analysis of the *ASR* Gene Family in the Rosaceae and Expression Analysis of *PbrASRs* During Fruit Development

**DOI:** 10.3389/fgene.2021.792250

**Published:** 2021-12-22

**Authors:** Biying Zhao, Xianrong Yi, Xin Qiao, Yan Tang, Zhimei Xu, Shanting Liu, Shaoling Zhang

**Affiliations:** ^1^ Guangxi Academy of Specialty Crops, Guilin, China; ^2^ Center of Pear Engineering Technology Research, State Key Laboratory of Crop Genetics and Germplasm Enhancement, College of Horticulture, Nanjing Agricultural University, Nanjing, China

**Keywords:** ASR, pear, gene expression pattern, fruit ripening, subcellular localization

## Abstract

The members of the Abscisic Acid (ABA) Stress and Ripening gene family (*ASR*) encode a class of plant-specific proteins with ABA/WDS domains that play important roles in fruit ripening, abiotic stress tolerance and biotic stress resistance in plants. The *ASR* gene family has been widely investigated in the monocotyledons and dicotyledons. Although the genome sequence is already available for eight fruit species of the Rosaceae, there is far less information about the evolutionary characteristics and the function of the *ASR* genes in the Rosaceae than in other plant families. Twenty-seven *ASR* genes were identified from species in the Rosaceae and divided into four subfamilies (I, II, III, and IV) on the basis of structural characteristics and phylogenetic analysis. Purifying selection was the primary force for *ASR* family gene evolution in eight Rosaceae species. qPCR experiments showed that the expression pattern of *PbrASR* genes from *Pyrus bretschneideri* was organ-specific, being mainly expressed in flower, fruit, leaf, and root. During fruit development, the mRNA abundance levels of different *PbrASR* genes were either down- or up-regulated, and were also induced by exogenous ABA. Furthermore, subcellular localization results showed that PbrASR proteins were mainly located in the nucleus and cytoplasm. These results provide a theoretical foundation for investigation of the evolution, expression, and functions of the *ASR* gene family in commercial fruit species of the Rosaceae family.

## Introduction

The adaptation of plants to various stressful environmental conditions are associated with changes in morphological traits, physiological and biochemical pathways, and expression of related genes to mitigate the damage caused by such biological and abiotic stresses. The ABA, Stress, and Ripening (*ASR*) genes encode a small, plant-specific, hydrophilic protein, which is involved not only in the response of plants to drought, high salt, low temperature, and abscisic acid stresses, but also in many plant metabolic processes, such as fruit development, maturation, and sugar metabolism (Iusem et al., 1993; Cakir et al., 2003; Saumonneau et al., 2012; Jia et al., 2016; Li et al., 2020). With the development of molecular biology methodologies and transcriptome sequencing technology, the first *ASR* gene was cloned and characterized from tomato (*Solanum lycopersicum* L.) in 1993 (Iusem et al., 1993), with further *ASR* gene members having subsequently been identified in, and cloned from, dicotyledons and monocotyledons. The number of genes in the *ASR* gene family are one in grape (Cakir et al., 2003), four in banana (Liu et al., 2010), six in rice (Gonzalez and Iusem, 2014), and ten in maize (Jeanneau et al., 2002). ASR proteins have also been reported from soybean, tea, and ginkgo (Shen et al., 2005; Li et al., 2013; Yue et al., 2017). Interestingly, no *ASR* gene family members have been reported in the model plant *Arabidopsis thaliana*.

Most ASR proteins have similar conserved physical characteristics, with the amino acid sequence having two highly conserved regions. The first is located in the N-terminus, with a sequence which is rich in histidine, whereas the second is located in the C-terminal DNA-binding domain, which is highly conserved, and which contains an ABA/water stress deficit (WDS) structural domain (Feng et al., 2016; Wang et al., 2016). The structural characteristics indicate that the ASR proteins may have an important function in plant response to dehydration and the abscisic acid signaling pathway. In addition, earlier research found that the C-terminus of ASR has a highly conserved nuclear localization sequence, which indicates that the ASR protein may be a nuclear-localized protein, possibly with transcription factor function (Hong et al., 2002). Later, Iusem *et al.* supported this hypothesis, with the ASR1 protein being localized in the nucleus of tomato (Iusem et al., 1993); additional studies have also shown that the ASR protein is located in the cytoplasm (Cakir et al., 2003) or the chloroplast (Arenhart et al., 2013). The expression characteristics of the *ASR* gene family differ among various species. For example, in tomato and grapefruit, *ASR* genes are mainly expressed in fruit and leaf (Maskin et al., 2001; Cakir et al., 2003), whereas *ASR* genes in maize and rice are mostly expressed in leaf and root (Arenhart et al., 2013; Li et al., 2018). Expression of different *ASR* genes in the same species also differ in response to abiotic stress and during the regulation of plant growth and development. For example, in tomato, *ASR1* was highly expressed during fruit ripening and water stress (Maskin et al., 2001), whereas *ASR2* expression was higher in roots and leaves in response to abiotic stress (Maskin et al., 2001). The expression levels of *SiASR3* and *SiASR4* were up-regulated in leaves of foxtail millet (*Setaria italica*) under drought stress, whereas *SiASR1*, *SiASR2*, *SiASR5*, and *SiASR6* were highly expressed in roots of the same plant under these conditions (Feng et al., 2016).

The function of *ASR* genes was initially shown to be related to plant growth and development, with particular relevance to fruit ripening. For example, the ASR protein could be detected in mature grapefruit juice, but not in the juice of immature grapefruits (Canel et al., 1995). The transcription of the *VvASR* gene in *Vitis vinifera* was highest before fruit setting and the color-change period of grapes, with its expression increasing slightly after the fruit ripening stage (Cakir et al., 2003). Overexpression of the *ASR* gene was associated with the early coloring of strawberry, as well as with increased concentrations of sugar and ABA in the fruit (Chen et al., 2011). Later research showed that overexpression of the *ASR* gene could also increase the ability of plants to tolerate abiotic stress. For example, overexpression of the wheat *TaASR1* gene in tobacco could increase the tolerance of the transgenic lines to drought stress and also play a key role in the antioxidant system to combat oxidative stress (Hu et al., 2013). In maize, *ZmASR1* plays an important role in maintaining yield under drought stress (Virlouvet et al., 2011). Overexpression of the plantain (*Musa paradisiaca*) *MpASR* gene in *Arabidopsis* increased the tolerance of the transgenic lines to osmotic stress (Dai et al., 2011), whereas overexpression of the foxtail millet *SiASR1* gene in *Arabidopsis* increased tolerance to drought and oxidative stress (Feng et al., 2016), and overexpression of an *ASR* gene from lily in *Arabidopsis* also resulted in increased tolerance to freezing stress (Hsu et al., 2011). In rice, the significantly improved cold tolerance achieved by overexpression of the *OsASR1* gene was associated with a higher photosynthetic rate (Kim et al., 2009). In addition, the *ASR* gene has also been implicated in resistance to biological stresses, with the expression levels of *MpASR* genes in plantain being up-regulated in root, leaf, and fruit peel in response to infection with the Fusarium wilt pathogen; when an *MpASR* gene was expressed in tobacco, resistance to Fusarium wilt disease was significantly improved (Liu et al., 2010). These results indicate that *ASR* genes also play a role in resistance to biotic stress.

The *ASR* gene family has been widely studied in both the monocotyledons and the dicotyledons, but the structure and function of members of the *ASR* gene family in the Rosaceae has not been researched in detail. The genome sequences of eight fruit species in the Rosaceae are already available, namely Chinese white pear (*Pyrus bretschneideri*), wild strawberry (*Fragaria vesca*), apple (*Malus* × *domestica*), peach (*Prunus persica*), black raspberry (*Rubus occidentalis*), Japanese apricot (*Prunus mume*), European pear (*Pyrus communis*), and sweet cherry (*Prunus avium*), which provides resources for further analysis of the *ASR* gene family in the Rosaceae. The present study aimed to screen for and identify all the *ASR* gene members, investigate their evolutionary history, selection pressure and drivers in the eight Rosaceae species, and explore the expression characteristics during fruit development in Chinese white pear. These results provide a valuable insight into understanding the evolutionary history and biological characteristics of *ASR* genes in the Rosaceae, and lay the foundation for further studies into the mechanisms of fruit development in the Rosaceae.

## Materials and Methods

### Whole-Genome Identification of *ASR* Genes in Members of the Rosaceae

For the identification of the *ASR* genes in plant species from the Rosaceae, multiple database searches were performed. The tomato, soybean, maize, wheat, foxtail millet, banana, and rice ASR protein sequences were downloaded from Phytozome v.13 (http://phytozome.jgi.doe.gov/pz/portal.html#). These sequences were used as queries to perform BLAST algorithm-based searches against eight Rosaceae genome databases. The Chinese white pear (*P. bretschneideri*, Nanjing Agricultural University, NJAU, v1.1) genome sequence was downloaded from the Pear Genome Project (http://peargenome.njau.edu.cn/). The apple (*M. domestica*, JGI, v1.1) and peach (*P. persica*, JGI, v2.1) genome sequences were downloaded from the Joint Genome Institute (JGI, http://www.jgi.doe.gov/). Genome sequences of European pear (*P. communis*, Genome database for Rosaceae, GDR, v1.1), black raspberry (*R. occidentalis*, GDR, v3.0), sweet cherry (*P. avium*, GDR, v1.0), and strawberry (*F. vesca*, GDR, v4.0) were obtained from the Genome database for Rosaceae (GDR, http://www.Rosaceae.org/). The Japanese apricot genome sequence was obtained from the *Prunus mume* (BUF, v1.0) Genome Project (http://prunusmumegenome.bjfu.edu.cn/index.jsp). The seed alignment file for the ASR domain (PF02496.16) was obtained from the Pfam database (http://pfam.xfam.org) and an HMM file was created using the HMMER3 software package. HMM searches were then performed against the local protein databases of the eight Rosaceae species. All candidate ASR protein sequences were checked using SMART (http://smart.embl-heidelberg.de/) and Pfam to verify the presence of ASR protein family domains, with the sequences lacking the ASR domain or redundant sequences being removed.

### Phylogenetic Analysis of the *ASR* Gene Family

The full-length amino acid sequences of the ASR proteins were aligned using MUSCLE, and the phylogenetic tree was constructed using the Neighbor-Joining method in MEGA6 (http://www.megasoftware.net/). Bootstrapping was carried out with 1,000 replications, and the Poisson correction distance model, p-distance, and the pairwise deletion option parameters in MEGA6 were selected.

### Structure and Conserved Sequence Analysis of the *ASR* Gene Family

The structures of *ASR* genes were studied using Gene Structure Display Server (GSDS 2.0) (http://gsds.cbi.pku.edu.cn/) by aligning the cDNA sequences with their corresponding genomic DNA sequences. The online Multiple Expectation Maximization for Motif Elicitation (MEME) (http://meme.nbcr.net/meme/cgibin/meme.cgi) tool was used to identify and analyze the conserved motifs of ASR proteins from the full-length amino acid sequences. In addition, the upstream regions (2000-bp upstream of the transcription start point) of the three *PbrASR* genes *PbrASR1*, *PbrASR2*, and *PbrASR3* were used to query the putative *cis*-acting elements in the PlantCARE database (Lescot et al., 2002).

### Chromosomal Localization and Syntenic Analysis of the *ASR* Gene Family

Information on chromosomal localization of *ASR* genes was acquired from each genome annotation file. A method similar to that developed for the Plant Genome Duplication database (PGDD) (http://chibba.agtec.uga.edu/duplication/) was then used to perform analysis of the syntenic relationships among the *ASR*s. Firstly, local all-vs-all BLASTP algorithm-based searches were conducted among the eight Rosaceae genomes to determine potential homologous gene pairs (E < 1e^−10^). Subsequently, MCScanX was employed for the identification of syntenic gene pairs, with the BLASTP result and gene location information as input documents (Wang et al., 2012). WGD, proximal duplication, tandem duplication, dispersed duplication, and transposed duplication events of *ASR* family genes were identified using the MCScanX package. Finally, the data were plotted using Circos software (Krzywinski et al., 2009); genes located on unanchored scaffolds are not shown.

### Calculation of Ka, Ks and Ka/Ks of the *ASR* Gene Family

The values of Ka, Ks, and the Ka/Ks ratio of homologous *ASR* gene pairs in the eight Rosaceae species were identified with KaKs_Calculator 2.0 (Wang et al., 2010). All homologous gene pairs and the coding sequence were collated, and multiple alignments were performed automatically using computing_Ka_Ks_pipe.pl script by MAFFT software, which were then converted to AXT format for submission to the KaKs_Calculator 2.0 in the GMYN model (Qiao et al., 2019). Subsequently, the readable results were acquired, including Ka, Ks, Ka/Ks ratio, and the p-distance.

### Total RNA Isolation and RT-qPCR Assays

In order to identify the expression patterns of *PbrASR* genes in different tissues, root, stem, and leaf tissues were harvested from two-year-old pear seedlings; flower, sepals, ripening fruits, ovaries, buds, and pollen grains were harvested from a six-year-old pear tree, and pollen tubes were collected from pollen grains cultured for 5 h. To further explore the expression patterns of *PbrASR* genes during fruit development in pear, fruit samples of “Dangshansuli” were harvested from eight-year-old trees at five different developmental stages (S1–S5). The first sampling was conducted on 22 April (S1), which corresponded to 15 days after flowering (DAF). Subsequent samples were harvested on 13 May (S2, 36 DAF), 27 June (S3, 80 DAF), 28 July (S4, 110 DAF), and 30 August (S5, 143 DAF). For ABA treatment, some pear fruits with no damage and uniform size at “S5” stage were dipped with 100 μM ABA for 2 minutes, and the samples were collected at different time points (0, 3, 6, 12 h) after treatment, then frozen in liquid nitrogen and stored at –80°C until use. Total RNA was extracted from each tissue sample using an RNA extraction kit (RNAsimple Total RNA Kit; Tiangen, Beijing, China), according to the manufacturer’s instructions. The first-strand cDNA was synthesized using M-MLV reverse transcriptase (Takara Bio, Shanghai, China). The primers for specific *PbrASR* genes and the housekeeping *Actin* gene were designed by Oligo 7 software (Supplementary Table S1), and the specificity of these primers was verified against the pear genome by the program Primer Search-Paired. RT-qPCR was performed by LightCycler SYBR Green I Master (Roche, Germany), and a 20 μl reaction mixture was adopted, including 10 μl LightCycler 480 SYBR Green I Master Mix (Roche, Basel, Switzerland), 2 μl of each primer (200 nM of each primer), 1 μl cDNA (80 ng), and 7 μl H_2_O. Three biological replicates and three technical replicates were performed for each qRT-PCR assay. The relative expression levels were calculated using the 2^–ΔΔCt^ method and normalized to that of the *PbrActin* gene, and the results were analyzed and graphed by Office 2010 (Microsoft Word for Mac) and GraphPad Prism 8 (Chen et al., 2020).

### Subcellular Localization of the *PbrASR* Genes

The amplification of full-length coding sequences of the *PbrASR* genes from Chinese white pear fruit and leaf tissue was carried out by PCR. The amplified PCR products were cloned directionally into the modified pCAMBIA1300-*GFP* plant expression vector, with the GFP reporter gene and the CaMV 35S promoter (Clontech, Beijing, China), manipulation of which resulted in the generation of PbrASRs-GFP. Primers for gene cloning and vector construction are shown in Supplementary Table S1. The recombinant plasmids, 35S-*PbrASR*s-*GFP*, as well as the control plasmid, 35S-*GFP*, were independently introduced into *Nicotiana benthamiana* leaves by *Agrobacterium*-mediated transformation, which was assessed according to a slightly modified version of the published method (Chen et al., 2019a). The GFP signal were visualized *via* the Zeiss LSM Image Browser (Zeiss, Germany) 3 days after transformation. Three independent transient expression assays were performed for each gene.

## Results

### Genome-Wide Identification and Classification of *ASR* Genes in the Rosaceae

**FIGURE 1:**
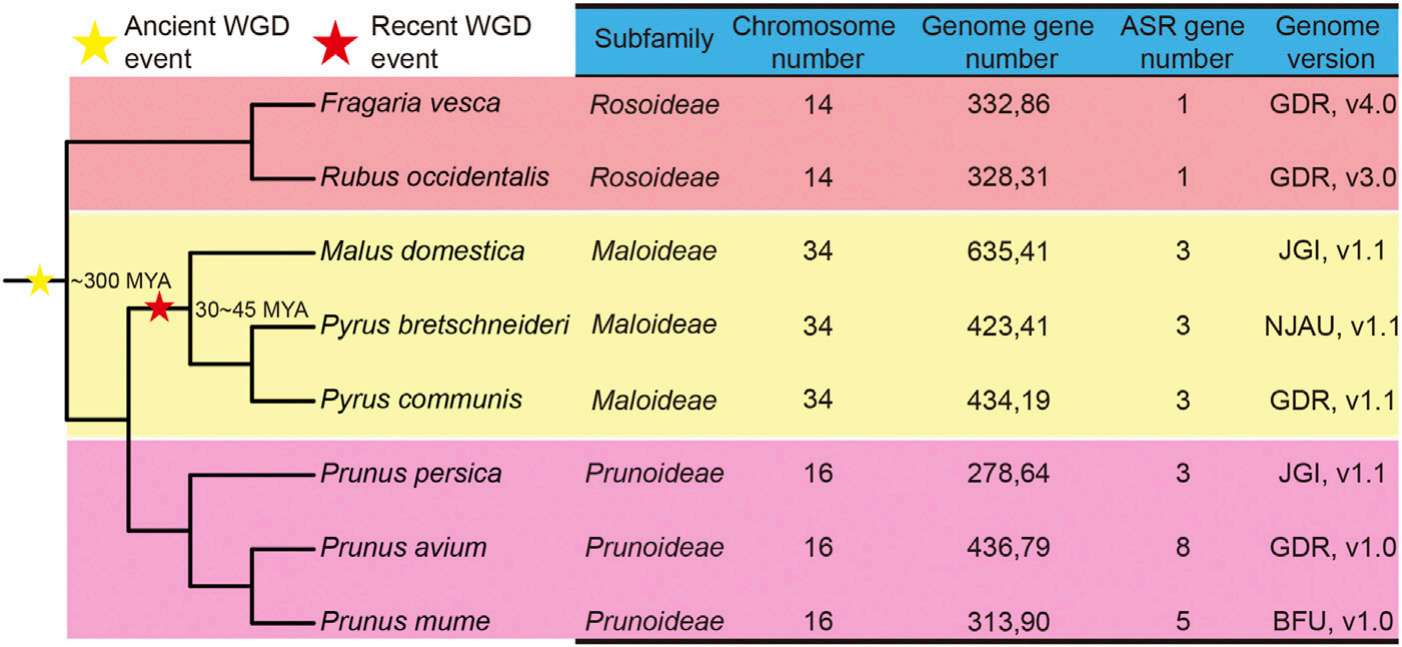
Phylogenetic tree and genome information on *ASR* genes in eight Rosaceae species. The stars indicate the divergent events in eight species. The yellow star represents the occurrence of an ancient whole-genome duplication (WGD) event, and the red star represents the occurrence of a recent WGD event. MYA: million years ago. The species tree was downloaded from NCBI Taxonomy common tree (http://www.ncbi.nlm.nih.gov/Taxonomy/CommonTree/wwwcmt.cgi), and the phylogenetic tree was constructed by MEGA6.

To identify the members of the *ASR* gene family in species of the Rosaceae, the amino acid sequences of ASR proteins from tomato were used as queries in the genomes of various species, while a Hidden Markov Model (HMM) search, using the *ASR* gene domain HMM profile (PF02496.16), was used to screen the Rosaceae genomes. Initially, a total of 36 *ASR* genes were identified in the Rosaceae, using these two strategies. Subsequently, transcripts of the same gene, redundant sequences, and incomplete gene sequences were removed. Ultimately, 27 non-redundant *ASR* genes were determined in the eight Rosaceae genomes, including three *ASR* genes identified from Chinese white pear (*PbrASR*s), one gene from strawberry (*FvASR*), three genes from apple (*MdASR*s), eight genes from sweet cherry (*PavASR*s), three genes from European pear (*PcoASR*s), five genes from Japanese apricot (*PmASR*s), three genes from peach (*PruASR*s), and one gene from black raspberry (*RocASR*) (Figure 1).

A phylogenetic tree of the eight Rosaceae species was constructed. Recent studies inferred that whole-genome duplication (WGD) events play an important role in the evolution of plant genes (Chen et al., 2020), mainly involving two events, namely the γ event (−300 million years ago, MYA) and the recent lineage-specific WGD event (30–45 MYA) (Wu et al., 2013) (Figure 1). Chinese white pear, apple, and European pear belong to the Maloideae subfamily; Japanese apricot, sweet cherry, and peach belong to the Prunoideae; and strawberry and black raspberry belong to the Rosoideae. As shown in Figure 1, two WGD events (recent and ancient) were experienced by species of the Maloideae, but only one ancient WGD event occurred in each of the Prunoideae and the Rosoideae. On the other hand, almost twice as many *ASR* genes were present in the Prunoideae species than in the Maloideae species, which indicated that the recent WGD did not cause the expansion of the *ASR* gene family in the Maloideae.

### Physicochemical Features of the ASR Proteins in the Rosaceae

In this study, we found that the lengths of the ASR protein sequences ranged from amino acids 66 to 311, with the molecular weights of these ASR proteins varying from 7,661.35 Da to 33,221.78 Da. The isoelectric point (pI) value of 96.3% of the ASR proteins was less than 7, which inferred that the ASR proteins from the eight Rosaceae fruit species are rich in acidic amino acids. (Chen et al., 2020). The results show that the grand average of hydropathicity index (GRAVY) values from all ASR proteins were negative (Table 1), indicating that all ASR proteins in the Rosaceae are hydrophilic [25]. In addition, we found that the highest and lowest aliphatic indices of ASR proteins were 82.6 and 17.67, respectively, with most ranging from 30 to 50, suggesting that these ASR proteins from the Rosaceae are thermostable (Table 1).

**TABLE 1:** Characteristics of the ASR proteins in Rosaceae.

Gene name	Subfamily	Protein length (aa)	Protein molecular weight (Da)	PI	GRAVY	Formula	Aliphatic index
Pbr030467.1 (PbrASR1)	I	203	22,353.75	5.63	−1.426	C961H1430N292O328S1	32.36
Pbr019476.1 (PbrASR2)	I	182	19,815.18	5.96	−1.387	C850H1274N264O285S2	33.41
Pbr019477.1 (PbrASR3)	II	134	15,285.64	6.1	−1.439	C671H1007N199O212S1	44.63
FvH4_2g13410.1 (FvASR1)	II	192	21,019.52	6.03	−1.278	C912H1355N267O306S2	33.7
MD05G1028100 (MdASR1)	I	202	22,125.46	5.74	−1.421	C953H1406N290O323S1	30.59
MD10G1029000 (MdASR2)	II	133	15,045.43	6.17	−1.276	C664H991N197O205S1	47.89
MD10G1029100 (MdASR3)	IV	119	13,512.67	9.69	−0.639	C603H975N181O166S3	82.86
Pav_sc0000863.1_g110.1.mk (PavASR1)	I	153	16,415.40	5.36	−1.082	C704H1042N210O244S2	41.57
Pav_sc0001830.1_g020.1.br (PavASR2)	III	283	29,989.79	5.39	−1.54	C1241H1845N405O470S1	17.67
Pav_sc0000863.1_g090.1.br (PavASR3)	III	277	29,351.20	5.53	−1.537	C1215H1811N399O457S1	18.05
Pav_sc0002659.1_g080.1.br (PavASR4)	III	269	28,063.26	5.53	−1.271	C1170H1766N374O433S2	28.03
Pav_sc0000863.1_g020.1.br (PavASR5)	III	276	29,150.83	5.13	−1.523	C1206H1783N391O460S1	18.12
Pav_sc0000863.1_g060.1.br (PavASR6)	IV	66	7,697.42	6.18	−1.432	C337H507N103O104S1	38.64
Pav_sc0002659.1_g070.1.br (PavASR7)	IV	66	7,730.45	6.24	−1.538	C337H510N104O105S1	37.12
Pav_sc0005261.1_g020.1.br (PavASR8)	IV	110	12,569.69	6.26	−1.328	C550H824N170O170S1	41
pycom05g02020 (PcoASR1)	I	202	22,051.46	5.7	−1.367	C950H1412N288O322S1	33.02
pycom10g01890 (PcoASR2)	II	134	15,271.62	6.1	−1.436	C670H1005N199O212S1	43.88
pycom10g01910 (PcoASR3)	II	135	15,461.92	6.48	−1.393	C685H1015N207O205S1	44.3
Pm027899 (PmASR1)	I	148	15,176.75	5	−1.105	C651H931N191O232S1	29.12
Pm001734 (PmASR2)	I	141	15,941.49	6.19	−1.293	C698H1064N214O216S1	51.99
Pm031043 (PmASR3)	III	223	23,848.04	5.66	−1.312	C1007H1505N315O360S2	28.07
Pm028905 (PmASR4)	IV	234	26,031.57	5.66	−0.978	C1113H1749N343O369S6	56.28
Pm028428 (PmASR5)	IV	66	7,661.35	6.2	−1.438	C335H499N105O102S1	38.64
Prupe.6G160200.1 (PruASR1)	I	98	11,027.03	6.4	−1.364	C474H729N149O153S2	40.82
Prupe.8G034100.1 (PruASR2)	I	193	20,759.86	5.68	−1.335	C893H1302N272O305S1	30.52
Prupe.8G034200.1 (PruASR3)	III	311	33,221.78	5.62	−1.374	C1399H2089N445O501S2	27.36
Ro02_G25954 (RocASR1)	II	192	21,091.48	5.77	−1.319	C913H1355N269O310S1	34.69

### Phylogenetic, Structural, and Conserved Motif Analysis of *ASR* Genes in the Rosaceae

**FIGURE 2:**
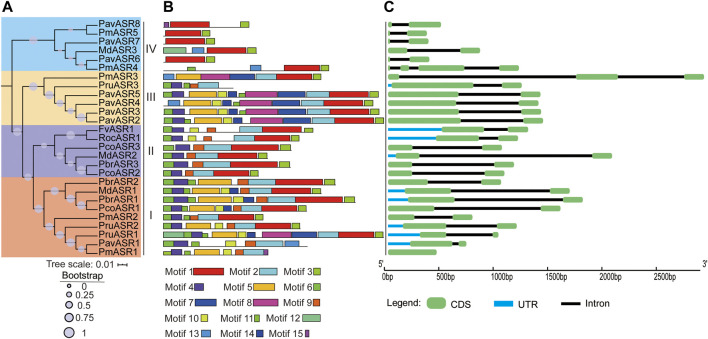
The phylogeny, conserved motifs, and structural analysis of *ASR* genes in eight Rosaceae species. **(A)** The phylogenetic tree was constructed by MEGA6, using the full-length amino acid sequences of ASR proteins with the Neighbor-Joining method and 1,000 bootstrap replicates. Different colors in the branches represent the different clades. The species in which ASR proteins were functionally characterized are displayed as icons. **(B)** Full-length amino acid sequences of ASR proteins of eight Rosaceae species were identified, using the MEME tool. In total, 15 motifs (Motifs 1–15), shown in different colors, are marked. The lengths and positions of the colored blocks correspond to the lengths and positions of motifs in the individual protein sequences. **(C)** The green boxes, black lines, and light blue boxes in the gene structural diagram represent exons, introns, and UTRs, respectively. Gene models are drawn to scale as indicated along the *x*-axis.

To classify the *ASR* genes and investigate their evolutionary relationships among the Rosaceae species, a phylogenetic tree was constructed, using the amino acid sequence analyzed by MEGA6 software. The results showed that the 27 *ASR* genes from the eight species were separated into four well-supported clades (Figure 2A). Of these, nine *ASR* genes were assigned to clade I, six were assigned to clade II, six were assigned to clade III, and six were assigned to clade IV. To further compare the evolutionary relationship of ASR proteins among Rosaceae species and ASR family members of other plants, a phylogenetic tree was constructed using the ASR protein sequences from rice, tomato, soybean, maize, wheat, foxtail millet, banana, and Rosaceae species (Supplementary Figure S1). The results showed that most of the ASR proteins in monocots and dicots were divided into different clusters, and a small part of ASR proteins were between them, which provided a theoretical basis for the possible functional differentiation of ASR proteins in the process of evolution.

To better understand the structural features of the *ASR* genes, exon/intron and conserved motif analyses were executed by aligning genomic sequences with their corresponding cDNAs, using the Gene Structure Display Server, and by discovering motifs from the full-length amino acid sequences of ASR proteins in the eight Rosaceae species, using the MEME tool, respectively. The result of the gene structure analysis showed that most individual *ASR* genes exhibited the same number of exons/introns, namely only two exons and one intron. For example, most *ASR* genes in clade I contain two exons, except for *PmASR1*, which had only one exon; the only *ASR* gene in clade II not to contain two exons was *PmASR3*, which had three exons, whereas the only *ASR* gene in clade IV not to contain two exons was *PmASR4*, which had four exons (Figure 2C).

In addition, the conserved motif analysis of ASR proteins showed that 15 conserved motifs were detected in proteins from the eight Rosaceae species, termed Motifs 1–15, and the number of motifs contained in the ASR amino acid sequences differed among the clades (Figure 2B). For example, the number of conserved motifs ranged from five to ten in clade I, with most of the proteins having 7–9 conserved motifs. Clade II contained 6–7 conserved motifs, whereas clade III *ASR* genes encoded more conserved motifs (7–12) than did the other clades. The lowest number of conserved motifs occurred in the ASR proteins in clade IV, ranging from two to four. Furthermore, Motif 1 and Motif 3 are basic regions of the ASR domain, and were identified in most members of the ASR protein family. Motifs 4, 6, 9, and 11 were detected in most members of clade I, and may be functional motifs common to genes involved in tolerance to abiotic stress. Motifs 1, 2, 3, 4, 6, and 9 were present in all members of clade II, while Motifs 1 and 3 were detected in all members of clade IV. Motif 7 was detected only in proteins of subgroup III, which may reflect its specific functions in this subgroup. In brief, the specificity and conservation of the number of motifs and exons in each clade support the close evolutionary relationship of *ASR* genes, indicating that members of the different clades may carry out different functions in different organs.

### The Evolutionary Expansion and Syntenic Analysis of *ASR* Genes in the Rosaceae

**FIGURE 3:**
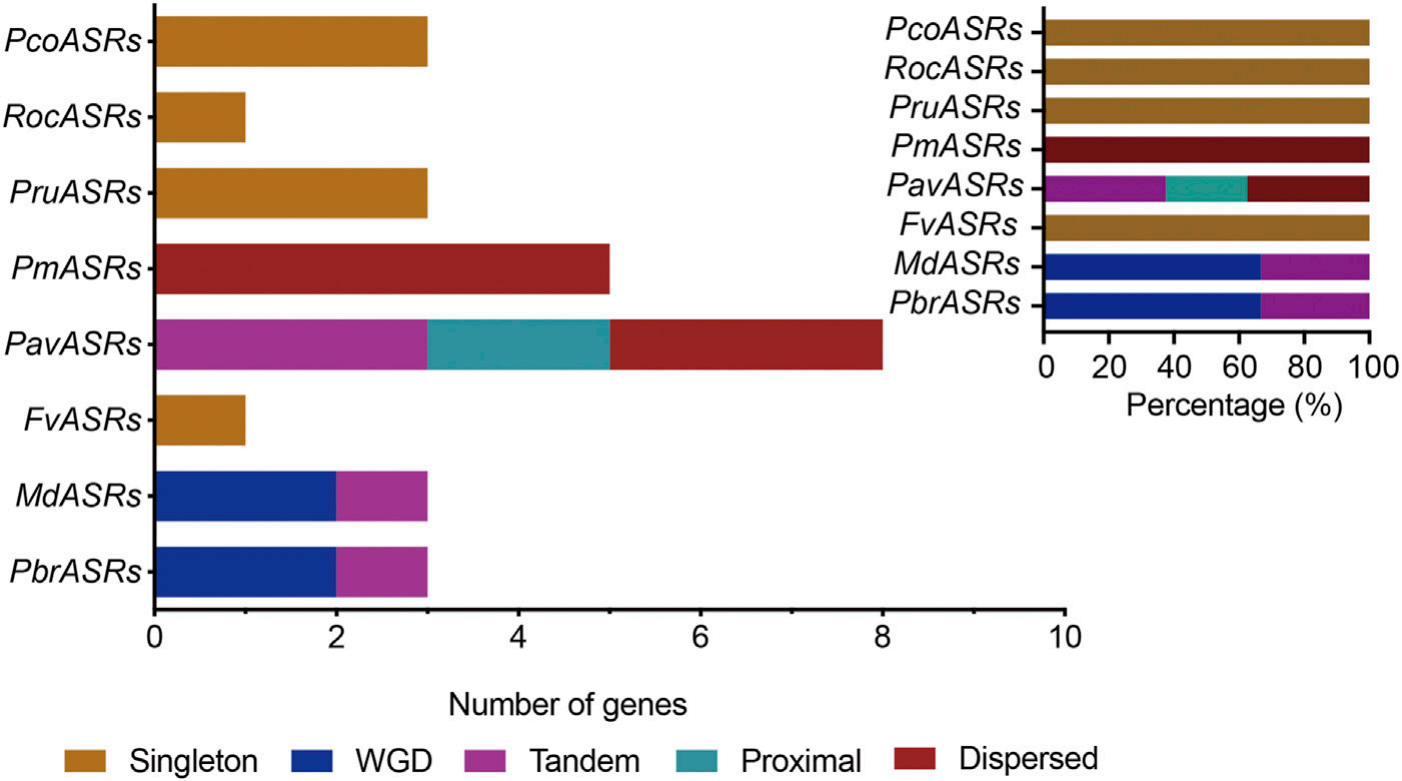
The number of *ASR* gene pairs evolved by different modes of gene duplication in eight Rosaceae species. The origins of duplicate *ASR* genes were determined by MCScanX and Prism 8 software. The *x*-axis in the lower histogram indicates the numbers of *ASR* genes. The *x*-axis of the upper-right histogram indicates the percentage of each duplicated event, including whole-genome duplication (WGD). The *y*-axis of both histograms indicates *ASR* genes from the different Rosaceae species. Different color bars represent different duplicated events.

The main driving force of evolution is gene duplication, which includes single-gene duplication events and whole-genome duplication events. To explore the evolutionary origins of the *ASR* gene family, evidence for the evolutionary occurrence in the *ASR* gene family of the different types of gene duplication, i.e., WGD, dispersed, tandem, proximal, and singleton, was investigated. The results of gene duplication analysis showed that 33.3% of *ASR* genes were derived from single-gene duplications in Chinese white pear, compared with 33.3% in apple, and 100% in each of peach, strawberry, Japanese apricot, black raspberry, cherry, and European pear (Figure 3; Supplementary Table S2). In contrast, genes derived from WGD events in the eight Rosaceae species were identified for only Chinese white pear and apple, with each having a relatively high proportion (66.7%) of *ASR* genes associated with WGD events (Figure 3; Supplementary Table S2). These results indicated that the expansion of *ASR* genes in the Rosaceae was not caused by the ancient WGD event, but may have been caused by the recent WGD event in apple and Chinese white pear. On the other hand, *ASR* genes experienced a high frequency of singleton duplication events (such as peach, strawberry, black raspberry, and European) and dispersed duplication events (such as Japanese apricot), which contributed to the expansion of the *ASR* gene family during evolution.

**FIGURE 4:**
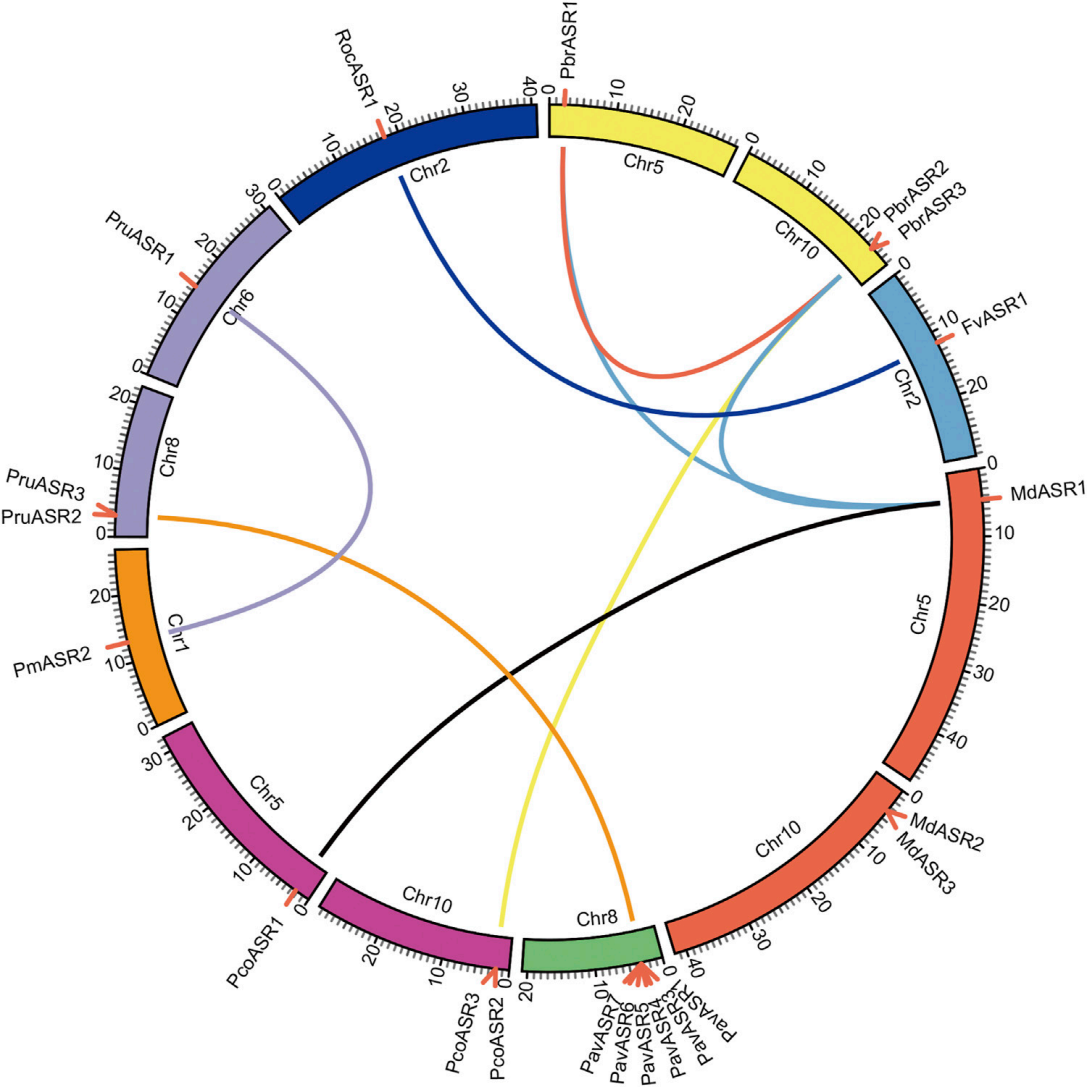
Chromosomal location of *ASR* genes and syntenic relationships among *ASR* genes in eight Rosaceae species. The circular forms of the chromosomes of the eight Rosaceae species are shown in different colors. The pink lines around the circumference of the circle mark the gene positions. The lines in different colors inside the circle represent collinearity relationships among the genes from the eight Rosaceae species.

To explore the evolutionary routes and understand the differences in diversity, both intra- and intergenomic syntenic analyses of the *ASR* genes were carried out among the eight Rosaceae species. The results showed that the three *PbrASR* genes were distributed on two of the 17 Chinese white pear chromosomes, with one *ASR* gene located on Chr5 and two ASR genes located on Chr10, with three syntenic pairs detected between intra- and intergenomic blocks; the three *ASR* genes were distributed on two of the 17 chromosomes in European pear, with two genes located on Chr10, and two syntenic pairs were identified among intra- and intergenomic blocks. The three *ASR* genes in apple were assigned to two of the 17 chromosomes, with two genes located on Chr10, and three syntenic pairs being detected among intra- and intergenomic blocks. Three *ASR* genes were distributed on two of the eight chromosomes in peach, with two genes located on Chr8, with two syntenic pairs identified among intra- and intergenomic blocks. Furthermore, only one syntenic pair was found in the other Rosaceae species, and their chromosomal distribution was assigned randomly (Figure 4).

### Ks Value and Ka/Ks Ratio Analysis of *ASR* Genes in the Rosaceae

**FIGURE 5:**
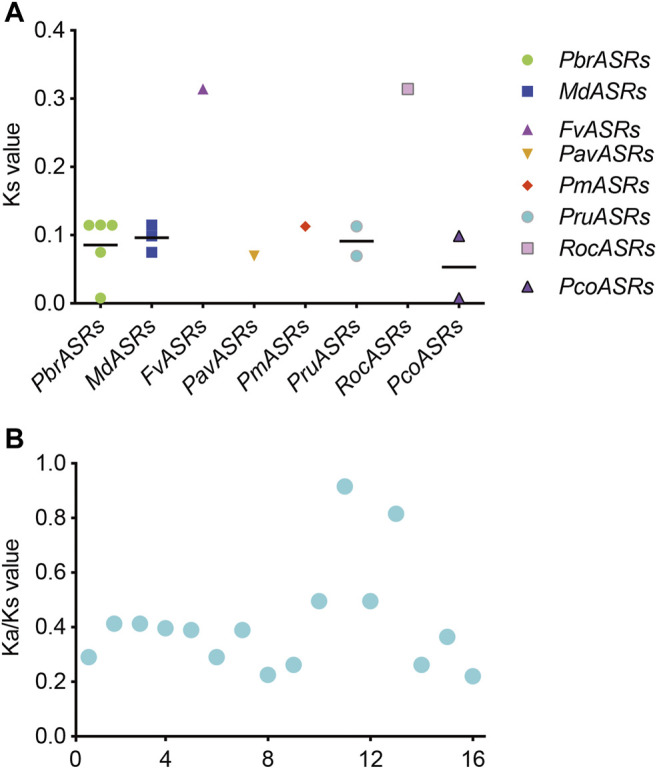
Distribution of average Ks and Ka/Ks values for *ASR* genes in eight Rosaceae species. **(A)** Ks values representing the times of *ASR* gene evolution in the Rosaceae. **(B)** Ka/Ks ratios representing the driving forces of *ASR* gene evolution in the Rosaceae.

The Ks value is commonly used to explore the approximate occurrence dates of WGD or segmental duplication events. Previous studies have shown that two WGD events had occurred during the genome evolution of apple and pear, including the recent WGD event (Ks ∼0.15–0.3 in pear, and Ks ∼0.2 in apple) that is inferred to have happened 30–45 MYA, and an ancient WGD event (Ks ∼1.5–1.8 in pear, and Ks ∼1.6 in apple) that is inferred to have occurred ∼140 MYA (Zhu et al., 2020). To explore the evolutionary dates of the WGD or segmental duplication events, Ks values of duplicated *ASR* gene pairs were analyzed in the Rosaceae species. The Ks values for eight *ASR* gene pairs in the syntenic region ranged from 0.00785 to 0.31413 (Figure 5A). The Ks values of the WGD duplicated gene pair *PbrASR1* and *PbrASR2* (Ks ∼0.11466) were close to the Ks peak corresponding to the recent WGD event in the Chinese white pear genome, which indicated that these *ASR* genes were derived and retained from a more recent WGD event, taking place 30–45 MYA (Wu et al., 2013). In addition, the Ka/Ks ratio has been extensively used as an index for investigating the direction and strength of selection pressures. For example, a value for the Ka/Ks ratio of less than one indicates purifying selection (negative selection), a value equal to one indicates neutral selection, and greater than one indicates Darwinian selection (positive selection) (Wu et al., 2013). Positive selection can select for advantageous mutations, and negative selection can remove deleterious mutations (Qiao et al., 2015). In this study, the results showed that the values of the Ka/Ks ratio for all *ASR* gene pairs in each species were less than one (Figure 5B), suggesting that negative selection was the primary driving force for evolution of the *ASR* gene family in the Rosaceae species.

### Expression Analysis of *PbrASR* Genes in Different Organs

**FIGURE 6:**
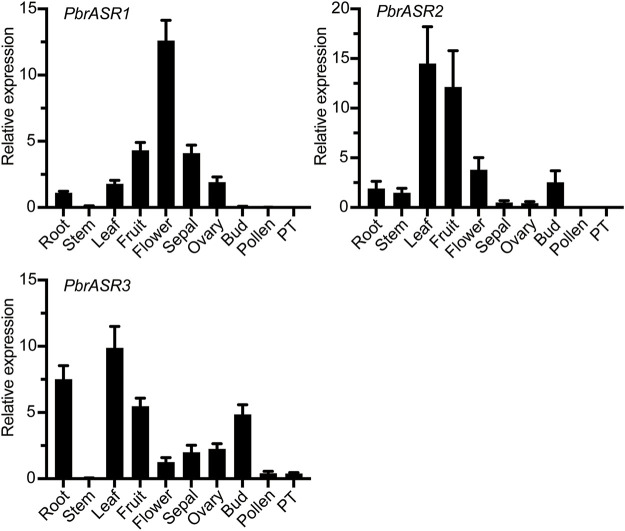
Expression pattern analysis of the three *PbrASR* genes in different organs. The relative expression levels of *PbrASR* genes were examined by RT-qPCR. Total RNA was extracted from root, stem, leaf, fruit, flower, sepal, ovary, bud, pollen, or pollen tubes (PT). For each gene, the relative expression levels were presented by normalization with expression of the pear *Actin* gene. The error bars indicate standard deviations, and data are summarized as mean ± standard deviation.

To identify the expression patterns and functional properties of the *ASR* genes in various organs in the Rosaceae, the *PbrASR*s from Chinese white pear was selected as candidate genes to characterize expression patterns by qPCR, using gene-specific primers (Supplementary Table S1). The organ-specific expression patterns of *PbrASR* genes were investigated in root, stem, leaf, fruit, flower, sepal, ovary, bud, pollen, and pollen tube. The results showed that most of the *PbrASR* genes were preferentially expressed in the leaf and fruit, suggesting largely organ-specific functions of the *PbrASR* genes. For example, *PbrASR1* exhibited a high expression level in flower tissues, indicating that this gene may have critical functions during flowering of Chinese white pear. *PbrASR2* was mainly expressed in leaf and fruit, with lower expression in flower, bud, and root. On the other hand, *PbrASR3* showed preferential expression levels in leaf, root, fruit, and bud, in addition to lower levels of expression in pollen and pollen tube (Figure 6).

### Effect of Exogenous ABA Treatment on *PbrASR’s* Gene Expression

**FIGURE 7:**
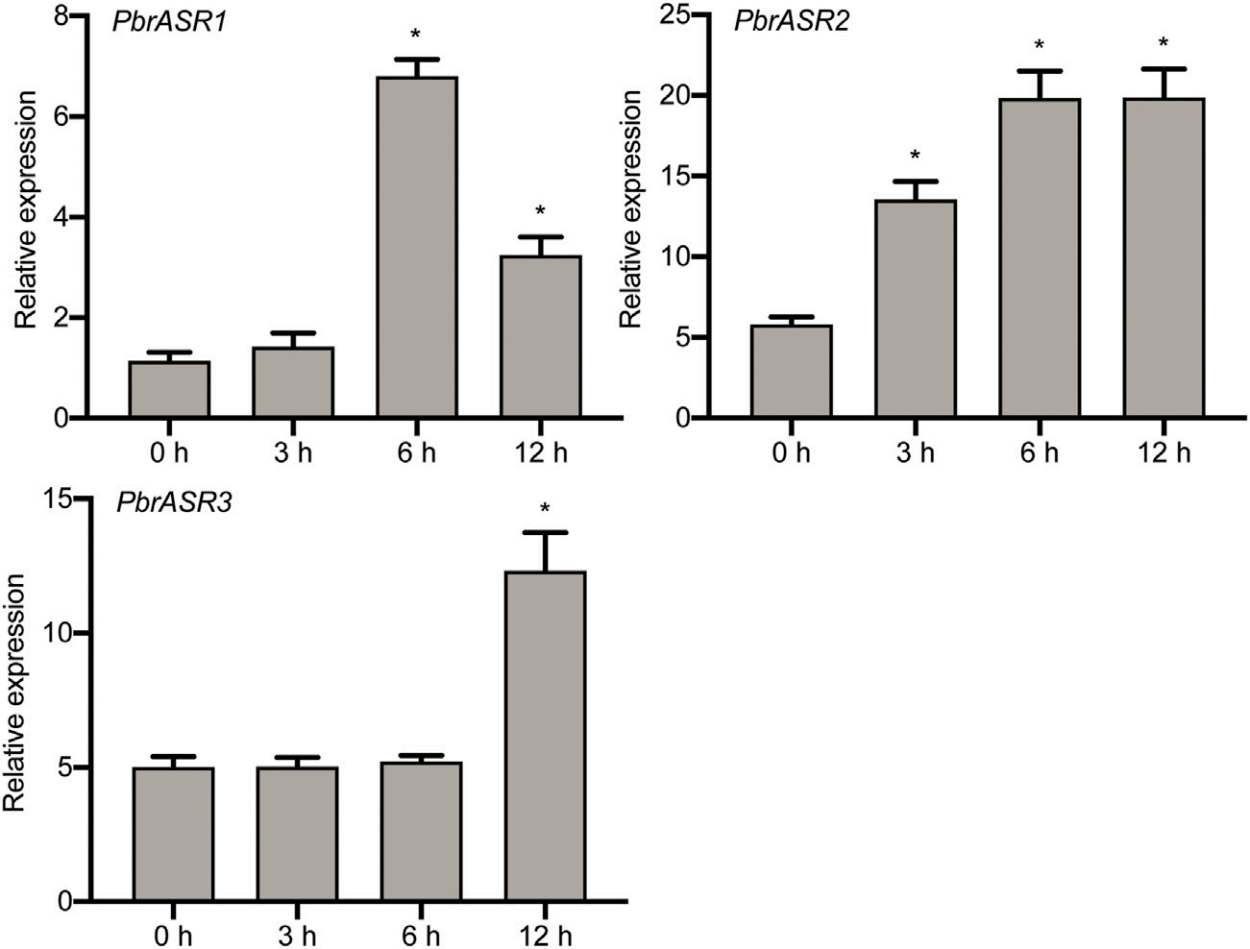
Expression levels of *PbrASR* genes following exogenous ABA treatment. Fruits sampled were collected at different time times (0, 3, 6, 12 h) after 100 μM ABA for 2 minutes, the relative transcript levels of *PbrASR* genes were analyzed by qRT-PCR. For each gene, the relative expression levels were acquired by normalization with expression of the pear *Actin* gene. The error bars indicate standard deviations. Data are summarized as mean ± standard deviation. Asterisks indicate a significant difference (*p < 0.05) compared with control at the different time points under exogenous ABA treatment.

To investigate the possible association of *PbrASRs* mRNA with exogenous ABA-accelerated pear fruit ripening, the expression of PbrASR genes were analyzed by qRT-PCR after application of exogenous ABA. The results indicated that the mRNA abundance levels of *PbrASRs* were induced by exogenous ABA treatment on the ‘S5’ stage pear fruit (Figure 7). For example, the expression level of *PbrASR1* increased by 5.9-fold after 6 h ABA treatment; *PbrASR2*’s mRNA transcripts began to increase within 3 h after ABA treatment, and rose to a peak at about 6 h. Furthermore, the expression level of *PbrASR3* gene was also increased under ABA treatment, and the highest expression level was observed at 12 h; Those results suggested that ABA treatment could induce the expression of *PbrASR* genes at transcriptional levels, and may play an important role in regulating fruit ripening.

### Possible Roles of *PbrASR* Genes in Fruit Development and Ripening

**FIGURE 8:**
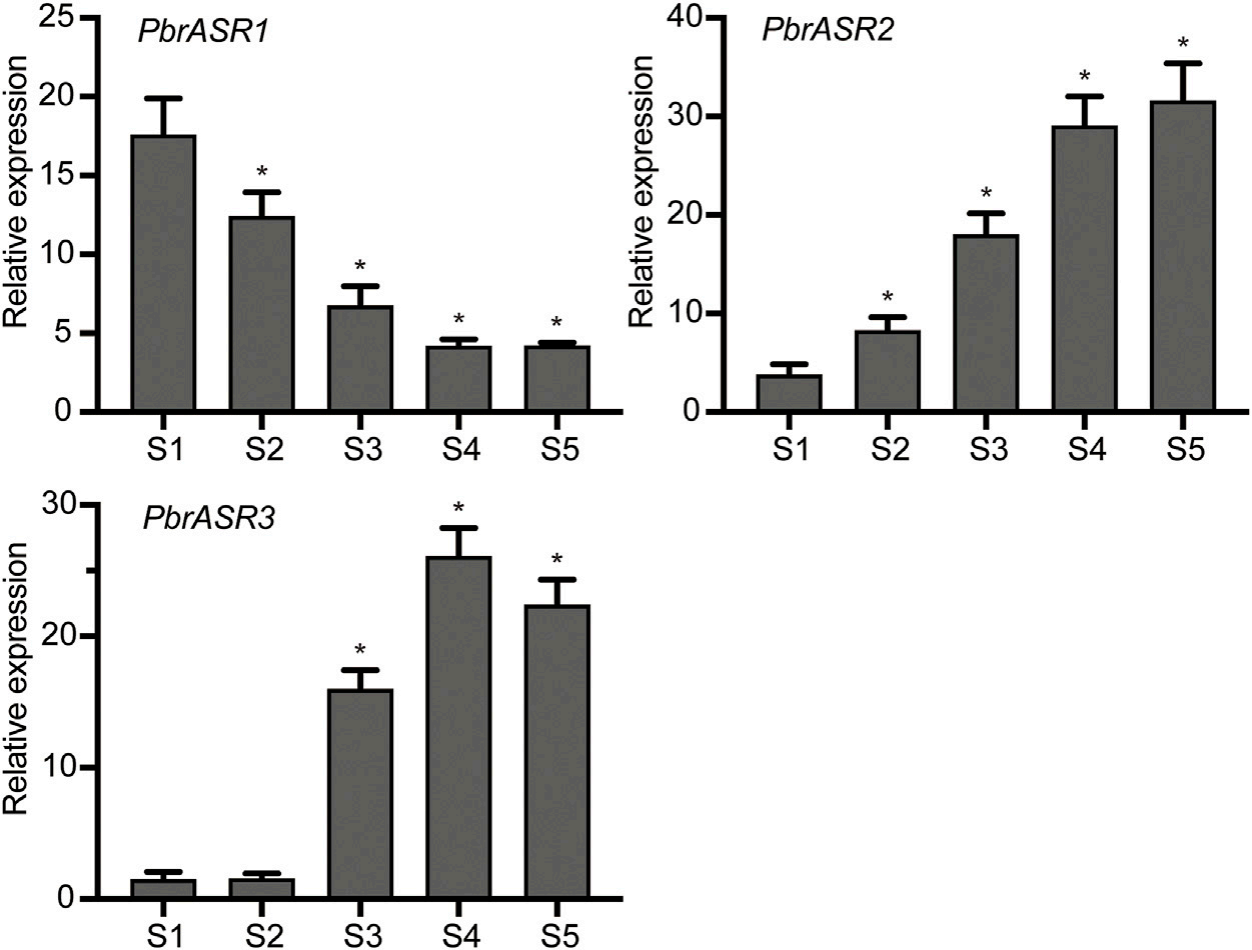
Expression values of *PbrASR* genes during fruit development. The relative transcript levels of *PbrASR* genes were analyzed by qRT-PCR, and the samples were harvested after flowering at S1, S2, S3, S4, and S5 in Chinese white pear from 8 year-old trees. For each gene, the relative expression levels were acquired by normalization with expression of the pear *Actin* gene. The error bars indicate standard deviations. Data are summarized as mean ± standard deviation. Asterisks indicate a significant difference (*p < 0.05) compared with S1 at the different time points during fruit development.

In order to evaluate the possible mechanism of regulation of *ASR* gene expression during fruit development and ripening, the promoters of three fruit-specific ASR genes were analyzed. The results showed that *cis*-acting elements were mainly divided into two categories: 1) involved in the perception of plant hormones, such as abscisic acid, methyl jasmonate, salicylic acid, auxins, and gibberellic acid; and 2) associated with responses to environmental and physiological stimuli, such as defense and stress responsiveness, drought inducibility, and light responsiveness (Supplementary Table S3). Therefore, it is postulated that *PbrASR* genes may be involved in the regulation of pear fruit development and ripening by influencing specific hormone signal transduction pathways. To determine whether the *PbrASR* genes were involved in fruit ripening, the mRNA abundance of individual genes was determined by qRT-PCR using the pear fruits sampled at five stages of development. The results showed that the mRNA abundance levels of *PbrASR1* significantly decreased from S1 to S4, but remained unchanged from S4 to S5 (Figure 8). For the *PbrASR2* gene, the pattern of its mRNA levels was opposite to that of *PbrASR1*, increasing from S1 to S4, with no change from S4 to S5 (Figure 8). The mRNA transcript levels of the *PbrASR3* gene did not vary significantly from S1 to S2, whereas, from S2 onward, the expression levels rapidly increased until S4, followed by a significant decrease to S5 (Figure 8). These results indicated that the expression levels of different *PbrASR* genes showed different patterns during pear fruit development and therefore these genes may be involved in the regulation of different fruit ripening responses.

### Subcellular Localization of *ASR* Gene Products in Chinese White Pear

**FIGURE 9:**
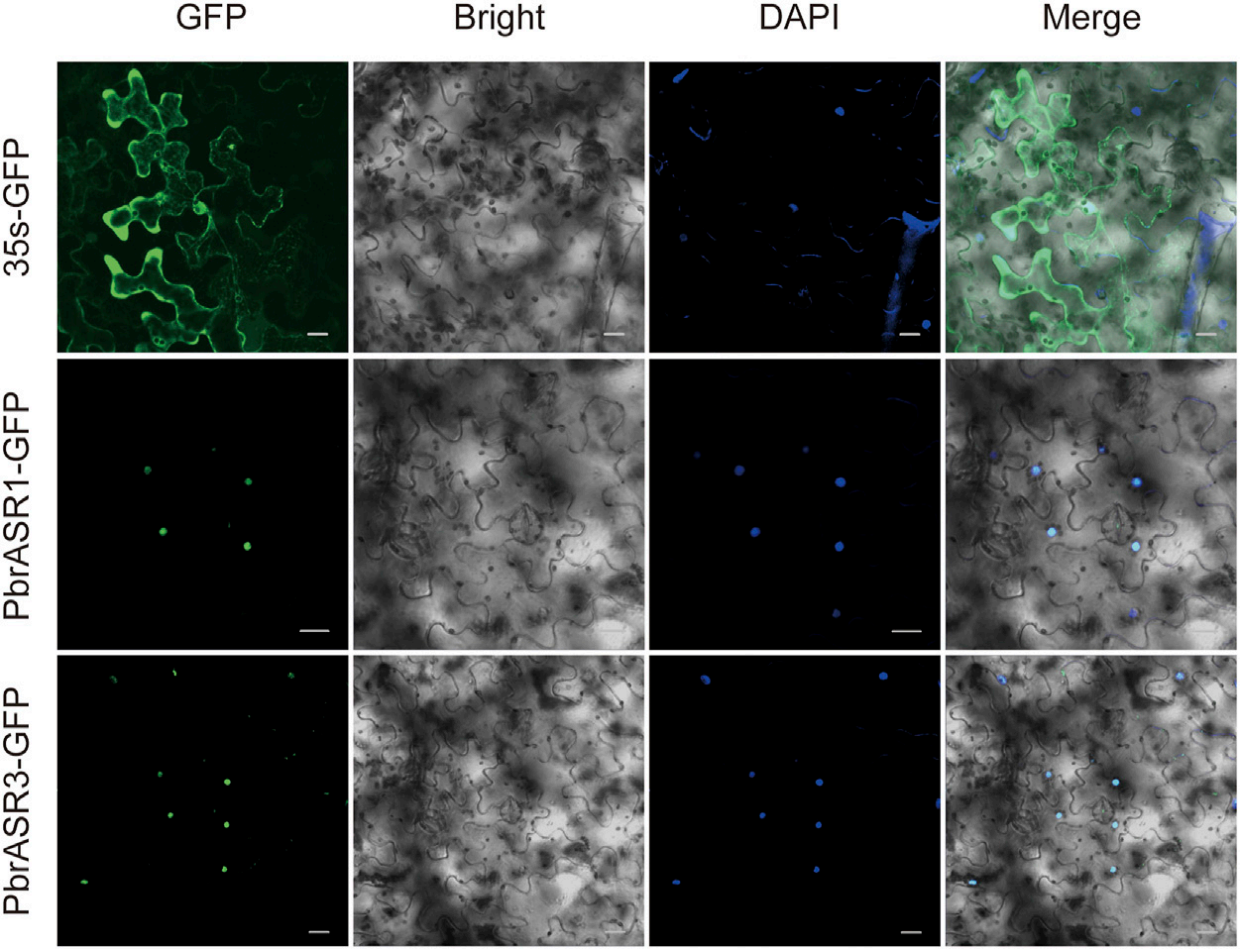
Subcellular localization of PbrASR proteins. Two *PbrASR* genes were cloned from Chinese white pear and used to construct CaMV35S:*ASR*-*GFP* vectors in which GFP was fused to ASR at the C-terminus. The three fusion proteins, with 35S-GFP as the control, were expressed transiently in tobacco (*Nicotiana benthamiana*) leaves in an independent manner and expression was observed and recorded under a laser confocal microscope. The merged images include the green fluorescence channel (first panels) and the nuclear dye DAPI in the blue fluorescence channel (third panels). The corresponding brightfield images are shown in the second panels. Bar = 20 μm.

To determine the subcellular locations of ASR proteins in Chinese white pear, structural analysis on PbrASR proteins was performed using the WoLF PSORT Subcellular Localization Prediction software. The sequence analysis showed that the ASR protein may be located in both the nucleus and the cytoplasm (Supplementary Table S4). To confirm these results, two *PbrASR* genes were cloned from leaf and fruit of Chinese white pear. The constructs encoding the ASR-green fluorescent protein fusion protein (PbrASR-GFP) and the control protein (35S-GFP) were transformed into *Nicotiana benthamiana* leaves. The results from laser confocal microscopy showed that the green fluorescence of the control (35S-GFP) was distributed throughout the whole cell, whereas the green fluorescence of the PbrASR1-GFP fusion protein was observed exclusively in the nucleus (Figure 9). Repeating the study with the PbrASR3-GFP fusion protein showed that it was located not only in the nucleus, but also in the cytoplasm (Figure 9). Therefore, the PbrASR1 protein exhibits a subcellular localization pattern in the nucleus, similar to that shown by the tomato SlASR protein, whereas PbrASR3 is also present in the cytoplasm as well as the nucleus, suggesting biological functions different from those of PbrASR1.

## Discussion

In order to adapt to the natural environment, a set of signal transduction pathways has evolved in plants to cope with responses to the complex and changeable environment. ASR proteins are small, hydrophilic plant-specific proteins that are widely involved in fruit ripening, sugar metabolism, and particularly in plant response to various biotic and abiotic stresses (Cakir et al., 2003; Liu et al., 2010; Saumonneau et al., 2012; Jia et al., 2016; Li et al., 2020). It is possible to comprehensively analyze the potential functions of a gene family from the genomic perspective as entire genome sequencing has been carried out on more and more plant species. The members of the *ASR* gene family have been investigated in many plant species, including tomato, banana, maize, and rice (Iusem et al., 1993; Jeanneau et al., 2002; Liu et al., 2010; Gonzalez and Iusem, 2014). However, only limited research on the *ASR* gene family has been carried out in the rosaceous species, especially in the increasingly important fruit crop Chinese white pear, where no research results have been published.

In the present study, 27 *ASR* genes were identified in eight members of the Rosaceae, with one copy in strawberry, five in Japanese apricot, one in black raspberry, eight in sweet cherry, and three in each of Chinese white pear, apple, European pear, and peach. Although two WGD events (a recent WGD event and an ancient WGD event) occurred in the Maloideae (Chinese white pear, European pear, and apple) (Qiao et al., 2015), and one WGD event (the ancient WGD event) happened in both the Prunoideae (peach, sweet cherry, and Japanese apricot) and the Rosoideae (black raspberry and strawberry) (Qiao et al., 2015), the WGDs did not lead to large-scale expansion of the number of *ASR* gene family members. These results suggested that the *ASR* gene family is small and evolutionarily conserved. Furthermore, these *ASR* genes from Rosaceae species were classified into four clades in the phylogenetic tree constructed, namely I, II, III, and IV, a finding that is consistent with the classification of *ASR* genes from other plant species (Li et al., 2020). Of these, six *ASR* genes were classified into each of clades IV, III, and II, with nine *ASR* genes classified into clade I. The results obtained from conserved motif and exon-intron analyses provided some clues regarding the evolution of the clades in the phylogenetic tree based on the *ARS* gene family (Zeng et al., 2020). In the present study, we found that most genes in clade IV contained between two and four conversed motifs and two exons (except for *PmASR4*), which may be the result of fragment loss during gene duplication. Members of clade III included 10–12 conserved motifs and two exons (except for *PmASR3*), clade II included 6–7 conserved motifs and two exons, and clade I included 7–10 conserved motifs (except for *PruASR1*) and two exons (except for *PmASR1*). These results indicate that the number of exons-introns and conserved motifs also supported the classification of the *ASR* genes in Rosaceae (except for *PmASR*s), with the *ASR* gene family having evolved various conserved motif organizations over its long evolutionary history, which might result in functional diversity among different clades of *ASR* genes.

To investigate the collinearity relationships and the evolutionary history of *ASR* genes in the Rosaceae, and to further explore their potential functions in terms of response to various biotic and abiotic stresses, and the mechanism of fruit ripening, the gene syntenic relationships, gene duplication events, and Ka/Ks values of *ASR* genes were analyzed in members of the Rosaceae. In this study, each of three *PbrASR* genes from four pairs of paralogous genes was found to be located on two chromosomes, Chr5 and Chr10. The members of *ASR* genes in other Rosaceae species studied were also randomly distributed on only one or two chromosomes. In addition, the results of the syntenic analyses suggested that the *ASR* genes in Chinese white pear and apple were derived primarily from a WGD event, whereas dispersed duplications were the major drivers of *ASR* gene member expansion in Japanese apricot. The *ASR* genes in peach, strawberry, black raspberry, and European pear were derived mainly from singleton duplication events, whereas, in cherry, tandem and dispersed duplications were the main drivers of *ASR* gene family expansion. These results indicated that the *ASR* gene is relatively highly conserved during the process of evolution, and it is not distributed onto more than two chromosomes. Previous studies have shown that the Ka/Ks ratio represents a measure of the direction and the magnitude of the selection pressure: Ka/Ks > 1 represents Darwinian (positive) selection, Ka/Ks = 1 represents neutral evolution, and Ka/Ks < 1 represents purifying (negative) selection (Qiao et al., 2015). The results from the current study showed that the Ka/Ks values for all paralogous genes were less than one, which indicated that purifying selection played an important role in the evolution of the genes in the *PbrASR* family.

Since Iusem et al. cloned and analyzed the first *ASR* gene in tomato (Iusem et al., 1993), more and more researchers have explored the various function of *ASR* genes in plants (Cakir et al., 2003; Gonzalez and Iusem, 2014; Li et al., 2018). Gene expression patterns and subcellular localization of the protein encoded can provide important clues for exploring the function of genes (Chen et al., 2019b). The RT-qPCR results showed that the different *PbrASR* genes in Chinese white pear exhibited diverse spatiotemporal expression patterns in various organs, with *PbrASR1* being mainly expressed in the flower, indicating that it was possibly involved in flower development, with response to stresses and carbohydrate metabolism during flowering being possible roles, according to the literature on *ASR*s from other species. On the other hand, *PbrASR2* and *PbrASR3* exhibited preferential expression in leaf and fruit, implying that these genes potentially participate in fruit ripening or involve various response to abiotic stress by ABA signaling in leaves. In other plants, different *ASR* expression patterns have been reported, such as *SiASR1* being mainly expressed in the root, and being shown to play an important role in responding to drought and oxidative stresses (Feng et al., 2016); *BdASR1* in *Brachypodium* was preferentially expressed in the leaf, and functioned by encoding a transcription factor which increases drought tolerance (Wang et al., 2016); and *FaASR* was highly expressed in fruit, and shown to be involved in strawberry fruit ripening (Chen et al., 2011).

To explore the role of the *PbrASR* genes in fruit ripening in Chinese white pear, we analyzed the mRNA abundance levels of different *PbrASR* genes during the fruit development process, using qRT-PCR. *ASR* genes have been suggested to play key roles in plant responses to developmental signals, such as fruit ripening and senescence. For example, Maskin and others indicated that the expression levels of *ASR2* gene in tomato fruit showed a trend of decreasing expression as the fruit ripened (Maskin et al., 2001). In strawberry, the mRNA abundance levels of the *FaASR* gene increased during fruit development (Chen et al., 2011). Furthermore, Jia and co-workers also demonstrated that the mRNA abundance levels of *ASR* genes in tomato and strawberry fruit were dramatically increased during fruit development (Jia et al., 2016). In the present study, the dynamics of *PbrASR* gene mRNA abundance were investigated in Chinese white pear during the fruit development process. The results showed that expression of the *PbrASR1* gene was downregulated during the fruit development process, whereas that of *PbrASR2* and *PbrASR3* was upregulated during the same process. These results indicated that the expression of specific *PbrASR* genes might play some specific functions in pear fruits during fruit ripening.

Furthermore, the subcellular localization experiment showed that the proteins encoded by *PbrASR* genes were also located in the nucleus or nucleus and cytoplasm, findings which were similar to the results obtained for the *ASR* gene in tomato and foxtail millet (Iusem et al., 1993; Feng et al., 2016). These results indicated that *PbrASR* gene-encoded proteins, which were nuclear and/or cytoplasmically localized, were mainly expressed in fruits and leaves, and may regulate fruit ripening and participate in normal growth and development in response to various biotic and abiotic stresses.

## Conclusion

In summary, a total of 27 *ASR* genes were identified and analyzed in eight Rosaceae species, including the three *ASR* genes from the Chinese white pear genome. According to the gene structure, conserved protein motifs, and phylogenetic relationships, the *PbrASR* genes were clustered into four well-supported clades (I, II, III, and IV), with all members of the ASR protein being small and hydrophilic. Syntenic analysis showed that purifying selection was the primary force during *ASR* gene family evolution among the Rosaceae species, and that they were highly conserved during the process of evolution. The relative expression of the three *PbrASR* genes, *PbrASR1*, *PbrASR2*, and *PbrASR3*, revealed that these genes were preferentially expressed in leaf, flower, fruit, and root, with the mRNA abundance levels of *PbrASR1* being down-regulated during fruit ripening, whereas expression of *PbrASR2* and *PbrASR3* was up-regulated during fruit ripening. Furthermore, subcellular localization indicated that the proteins PbrASR1 and PbrASR3 were located in the nucleus, suggesting a possible role fro these ASR proteins as transcription factors. The data collected in this study establish a foundation for further investigations to evaluate the structures and functions of members of the *ASR* gene family in commercially important plants of the Rosaceae.

## Data Availability

The datasets presented in this study can be found in online repositories. The names of the repository/repositories and accession number(s) can be found in the article/Supplementary Material.

## Abbreviations

ASR, ABA-stress-ripening; WGD, Whole-genome duplication; ABA, Abscisic acid; PGDD, Plant genome duplication database; RT-qPCR, Quantitative real-time PCR; PT, Pollen tube; HMM, Hidden Markov Model.
