# Supplementary figures and images for: Genome-Wide Identification and Comparative Analysis of the ASR Gene Family in the Rosaceae and Expression Analysis of PbrASRs During Fruit Development
Source: Front Genet. 2021 Dec 22;12:792250. doi: 10.3389/fgene.2021.792250 (PMC8727533; doi:10.3389/fgene.2021.792250)

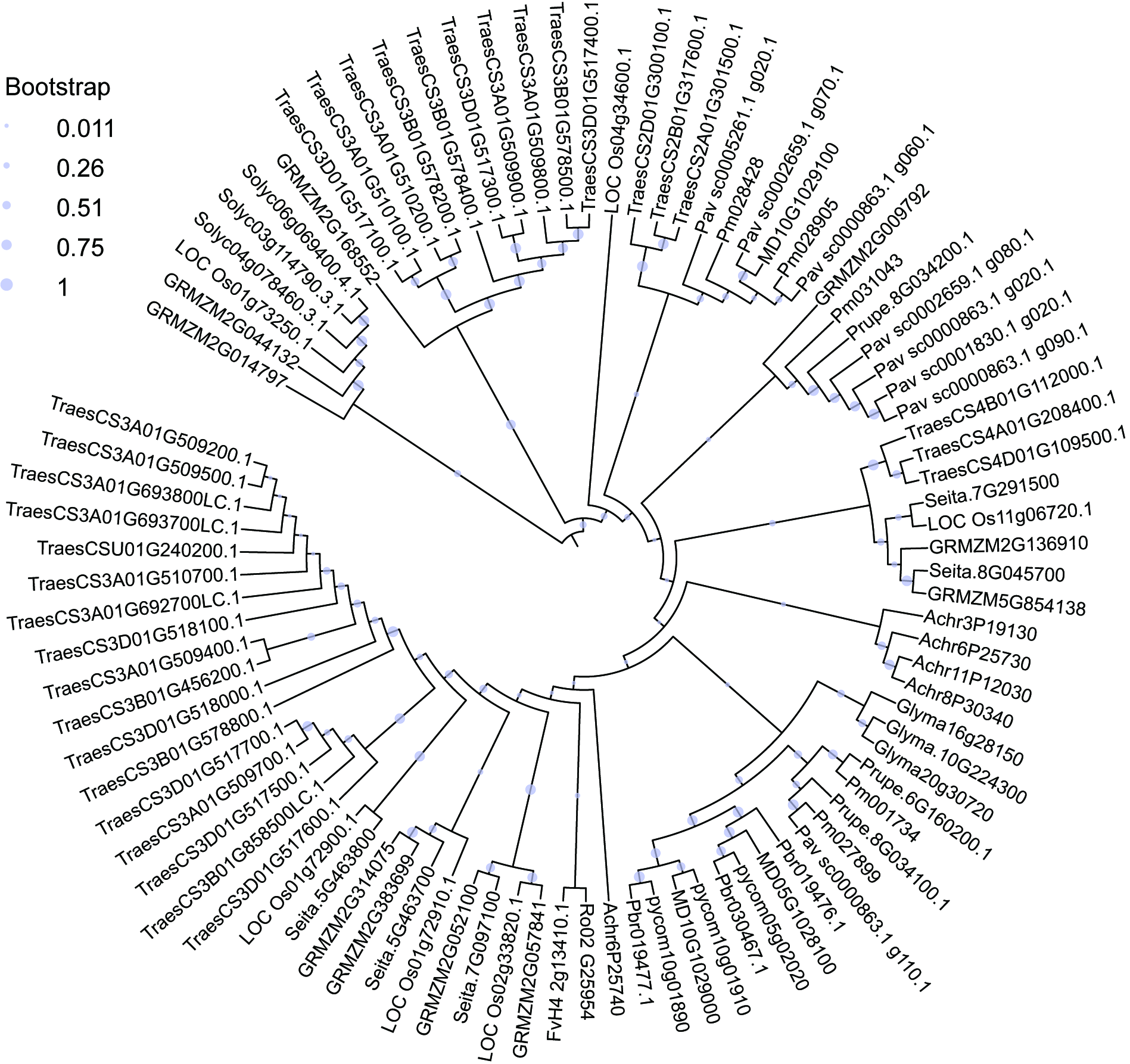

Supplement: Supplementary file 3 [file Image1.TIF]
